# Adolescent *Helicobacter pylori* Screening for Gastric Cancer Prevention: Current Evidence and Future Perspectives

**DOI:** 10.3390/children13091191

**Published:** 2026-09-04

**Authors:** Toshihiko Kakiuchi, Masumi Okuda, Hiroyoshi Endo, Masafumi Oka, Yasuhisa Sakata, Kazuma Fujimoto, Motohiro Esaki

**Affiliations:** 1 Departments of Pediatrics, Faculty of Medicine, Saga University, Saga 849-8501, Japan; 2 Department of Pediatrics, Hyogo Medical University, Nishinomiya 663-8501, Japan; 3 Department of Internal Medicine, Saiseikai Karatsu Hospital, Karatsu 847-0852, Japan; 4 Division of Gastroenterology, Department of Internal Medicine, Faculty of Medicine, Saga University, Saga 849-8501, Japan; 5 Graduate School of Medicine, International University of Health and Welfare, Okawa 831-8501, Japan

**Keywords:** *Helicobacter pylori*, adolescent screening, gastric cancer prevention, school-based screening, eradication therapy, antimicrobial resistance, care cascade, Japan

## Abstract

**Highlights:**

**What are the main findings?**

- Japanese adolescent *H. pylori* screening is feasible, but participation, diagnostic completion, antimicrobial resistance, and eradication outcomes vary substantially across programs.
- Current evidence supports early eradication biologically, but no trial has yet shown that adolescent screening reduces future gastric cancer incidence or mortality.

**What are the implications of the main findings?**

- Screening should be evaluated as a complete care cascade, including confirmation, treatment, eradication success, adverse events, resistance, and follow-up.
- As *H. pylori* prevalence declines, target populations, diagnostic pathways, treatment strategies, and cost-effectiveness should be periodically reassessed rather than fixed.

**Abstract:**

*Helicobacter pylori* infection is a major cause of gastric cancer, and eradication before advanced precancerous gastric changes develop provides a biologically plausible opportunity for primary prevention. Adolescence represents a strategically favorable, although not universally established, window for intervention in Japan because persistent childhood-acquired infection can be identified before extensive mucosal damage while school-based programs provide organized access to defined birth cohorts. This narrative review evaluates the evidence supporting population-based adolescent *H. pylori* screening, its potential harms and uncertainties, and the lessons derived from Japanese implementation. Representative programs demonstrate that organized screening is feasible but show substantial heterogeneity in participation, diagnostic pathways, antimicrobial resistance, eradication outcomes, and retention across the care cascade. Direct evidence that adolescent screening reduces future gastric cancer incidence or mortality is not yet available. As *H. pylori* prevalence declines, the balance among screening yield, diagnostic performance, antimicrobial exposure, cost, and equity will continue to change. International experience further indicates that prevention strategies should be matched to local epidemiology and healthcare systems. Adolescent screening should therefore be viewed as an adaptive prevention strategy whose target population, diagnostic pathway, and treatment approach require periodic reassessment.

## 1. Introduction

*Helicobacter pylori* (*H. pylori*) infection is the principal infectious cause of gastric cancer. Gastric cancer remains a major global health burden, and its incidence is particularly high in Eastern Asia [1]. Meanwhile, the global prevalence of *H. pylori* has declined substantially over recent decades, with lower prevalence reported in younger populations and higher-income settings [2]. These changing epidemiological conditions have important implications for the design and sustainability of population-based gastric cancer prevention strategies.

*H. pylori* infection is typically acquired during childhood [3] and usually persists unless successfully eradicated. Persistent infection produces chronic active gastritis that may progress over decades through gastric atrophy, intestinal metaplasia, dysplasia, and ultimately gastric cancer [4]. Randomized trials and meta-analyses demonstrate that eradication reduces subsequent gastric cancer risk, with evidence suggesting greater preventive benefit when treatment is undertaken before advanced precancerous lesions are established [5,6].

Adolescence represents a strategically attractive, although not universally established, window for intervention. Gastric mucosal injury can already be detected in infected children and adolescents, whereas advanced or extensive precancerous changes remain uncommon [7]. Clinically manifest gastric cancer is exceptionally rare during childhood and adolescence in Japan [8]. In addition, compulsory education provides a practical platform for organized screening and follow-up. These considerations are reflected in the 2024 revised Japanese guidelines, which address public-health testing of asymptomatic adolescents aged 12 years or older and identify junior high school as a practical timing for test-and-treat screening [9].

Japan has accumulated extensive real-world experience with school- and municipality-based adolescent screening. A recent review identified substantial heterogeneity among Japanese programs in target age, diagnostic pathways, treatment strategies, and implementation outcomes [10]. These programs demonstrate that organized screening is feasible, but feasibility and short-term eradication outcomes must be distinguished from the anticipated long-term effect on gastric cancer incidence, which has not yet been directly demonstrated in adolescent screening cohorts.

This review focuses specifically on population-based *H. pylori* screening during adolescence. We examine the biological and public-health rationale for this timing, evaluate the current evidence and its limitations, summarize representative Japanese implementation experience, and consider how future screening strategies should adapt to changing epidemiology, antimicrobial resistance, and healthcare-system conditions.

## 2. Why Adolescence Represents a Strategic Window for Population-Based *H. pylori* Screening

### 2.1. Biological Basis for Early Intervention

*H. pylori* infection is usually acquired during childhood and, once established, generally persists unless successfully eradicated [3]. Persistent infection initiates chronic gastric inflammation that may progress through the multistep sequence of atrophy, intestinal metaplasia, dysplasia, and gastric cancer [4]. This prolonged natural history creates a long interval during which preventive intervention is possible.

The rationale for early eradication is therefore not chronological age alone, but treatment before cumulative inflammatory injury and precancerous mucosal change become advanced. Long-term randomized and pooled evidence indicates that eradication reduces gastric cancer risk, and the 26.5-year follow-up of a randomized trial showed a particularly strong preventive association among participants without precancerous lesions at baseline [5,6]. These adult data do not establish the optimal age for adolescent screening, but they provide an important biological rationale for intervention before advanced mucosal injury.

### 2.2. Adolescence as a Period of Limited Precancerous Mucosal Damage

Gastric mucosal injury can already be present in *H. pylori*-infected children and adolescents, but the extent of precancerous remodeling is generally limited. In a Japanese pediatric cohort, moderate-to-marked gastric atrophy was observed in a minority of infected children, and intestinal metaplasia was not identified among children with grade 2–3 atrophy [7]. These findings argue against describing the adolescent stomach as unaffected, but they support the view that advanced precancerous change is uncommon at this age.

This pathological context is relevant to prevention. Adolescence may represent a stage at which persistent infection and chronic gastritis are already established while extensive irreversible mucosal remodeling remains relatively uncommon. Accordingly, eradication during adolescence is biologically plausible as an early intervention, although the magnitude of any lifetime cancer-preventive benefit cannot be determined from pediatric histology alone.

### 2.3. Timing Matters: Why Adolescence Rather than Young Adulthood?

The optimal age for population-based *H. pylori* screening has not been definitively established and is likely to depend on regional epidemiology, gastric cancer risk, and healthcare infrastructure. Current pediatric recommendations should therefore be interpreted within their geographical context. The updated ESPGHAN/NASPGHAN guideline, developed for the European and North American setting, does not support population-based screening of children from groups at increased gastric cancer risk solely on the basis of that background, while it supports noninvasive testing in children with a first-degree relative with gastric cancer [11].

In contrast, the revised Japanese guideline explicitly addresses adolescent test-and-treat strategies and recommends earlier eradication for gastric cancer prevention; it identifies junior high school as a practical timing while acknowledging the limited evidence defining a universally optimal screening age [9]. Adolescent screening in Japan should therefore not be interpreted as evidence that screening in young adulthood is ineffective. Rather, it is a context-specific strategy in which early biological intervention coincides with organized access to a defined population.

### 2.4. Public-Health Advantages of School-Based Screening

The rationale for adolescent screening extends beyond biological timing. Compulsory education provides a platform through which screening can be offered broadly without relying on individual healthcare-seeking behavior. The Japanese guideline emphasizes practical considerations such as access, parental involvement, and the feasibility of delivering screening within school-linked systems [9].

School-based programs also permit coordination among municipalities, schools, laboratories, and healthcare providers, potentially linking initial screening to confirmatory diagnosis, eradication therapy, and post-treatment assessment. However, high initial participation does not ensure completion of subsequent steps. Recent Japanese programs show that attrition may occur after a positive screening result, during confirmatory testing, or before treatment completion, making the integrity of the entire care cascade a central determinant of program performance [10].

### 2.5. Why Junior High School Students?

In Japan, many municipality-based programs have targeted students in junior high school, commonly at approximately 13–15 years of age [9,10]. This timing is not based on proof that a single biologically optimal age exists. Rather, it reflects a balance among the natural history of childhood-acquired infection, the rarity of gastric cancer before adulthood in Japan [8], the generally limited extent of precancerous mucosal damage during adolescence [7], and the logistical advantages of compulsory education.

Screening before students leave a common educational framework also allows adolescents and their parents to participate together in decisions regarding confirmatory testing and eradication therapy. Taken together, adolescence should be viewed not as a universally optimal age for screening but as a strategic window in which biological opportunity and population-level feasibility converge in the Japanese setting.

## 3. Evidence Supporting Adolescent Screening—And Its Limitations

### 3.1. Evidence That H. Pylori Eradication Reduces Gastric Cancer Risk

Randomized trials and meta-analyses provide substantial evidence that *H. pylori* eradication reduces the subsequent risk of gastric cancer, although most data are derived from adults [5,6]. In the randomized trial with 26.5 years of follow-up, eradication significantly reduced gastric cancer incidence, with a larger preventive effect among participants who did not have precancerous gastric lesions at baseline [6]. These findings support the principle that the timing of eradication relative to mucosal damage matters.

Eradication also reduces metachronous gastric cancer after endoscopic treatment of early gastric neoplasia. Randomized trials from Japan and Korea showed significantly lower rates of metachronous gastric cancer after successful eradication [12,13]. These populations differ fundamentally from asymptomatic adolescents, but the trials demonstrate that *H. pylori* remains a modifiable carcinogenic exposure even after substantial mucosal injury has occurred.

Importantly, these studies establish the cancer-preventive effect of eradication, not the effectiveness of population-based adolescent screening itself.

### 3.2. From Eradication Efficacy to Adolescent Screening: An Important Evidence Gap

No randomized trial has yet demonstrated that screening and eradicating *H. pylori* during adolescence reduces gastric cancer incidence or mortality later in life. Such evidence is inherently difficult to obtain because several decades may separate adolescent infection from clinically manifest gastric cancer. The rationale for adolescent screening therefore rests on converging rather than direct evidence: infection is acquired during childhood [3]; gastric carcinogenesis develops over decades [4]; advanced precancerous change is generally uncommon in children and adolescents [7]; eradication reduces later gastric cancer risk in adults [5,6]; and organized Japanese programs have shown that infected adolescents can be identified and treated within school-linked systems [10].

Accordingly, current evidence supports adolescent screening as a biologically plausible and operationally feasible prevention strategy in selected settings, while its long-term effect on gastric cancer incidence remains unestablished.

### 3.3. Diagnostic Strategy for Population-Based Adolescent Screening

Diagnostic methods used in population screening should be distinguished from those used for individual clinical diagnosis. Representative Japanese programs have commonly adopted a two-step strategy in which a noninvasive test suitable for large-scale school-based collection is used for initial screening, followed by confirmation of infection before treatment [14,15,16,17,18,19]. Urinary anti-*H. pylori* antibody testing has been frequently used for primary screening. Positive results have then been assessed using a stool antigen test or 13C-urea breath test, depending on the program [14,15,16,18,19].

The Matsumoto program used a more intensive pathway in which students with positive urinary antibody tests were offered endoscopy, histological assessment, and bacterial culture, allowing direct characterization of gastric mucosal changes and antimicrobial susceptibility [17]. These differences emphasize that no single diagnostic pathway has been uniformly adopted across Japan; algorithms have been shaped by local infrastructure and program objectives.

### 3.4. Screening Positivity and Confirmed Infection

A positive primary screening test should not be equated with confirmed active *H. pylori* infection. The distinction becomes increasingly important as prevalence declines because the positive predictive value of any screening test depends partly on the underlying prevalence of infection. Japanese programs therefore generally require a confirmatory assessment before treatment [9,14,15,16,17,18,19].

Yokosuka provides a clear example: 266 of 6270 screened students had a positive urinary antibody test, whereas 73 students were ultimately confirmed to have infection after secondary testing [19]. Reporting primary screening positivity and confirmed infection separately is essential when comparing programs and when evaluating how diagnostic pathways should evolve in lower-prevalence populations.

### 3.5. Eradication Therapy and Antimicrobial Resistance

Once infection is confirmed, treatment strategy should account for antimicrobial resistance, previous antibiotic exposure, and age-appropriate safety considerations [9,11]. Clarithromycin resistance is particularly relevant because it compromises clarithromycin-containing triple therapy. Japanese adolescent programs have consequently modified eradication strategies over time, including the use of vonoprazan-based regimens and susceptibility-guided treatment [15,17,18].

Program-specific eradication regimens and outcomes vary substantially and are summarized in Table 1. These differences should not be interpreted as head-to-head comparisons because the programs differed in study period, treatment selection, resistance patterns, and outcome definitions.

### 3.6. Safety Considerations

Eradication therapy is generally well tolerated in adolescents, but adverse events are not negligible when treatment is offered to otherwise asymptomatic individuals. Across Japanese programs, most reported adverse events were mild, but occasional events requiring medical attention occurred [14,15,17,18,19]. Safety should therefore be measured directly within screening programs rather than inferred solely from adult eradication studies.

Beyond immediate clinical adverse events, population-based antibiotic exposure raises broader concerns regarding antimicrobial resistance and perturbation of the gut microbiota. These ecological considerations are discussed in Section 4.

## 4. Balancing Benefits and Potential Harms of Population-Based Adolescent Screening

### 4.1. Potential Benefits and the Risk of Unnecessary Intervention

Population-based adolescent screening offers the possibility of identifying persistent *H. pylori* infection before advanced mucosal damage develops and before individuals leave an organized school-based framework. However, the strategy also exposes asymptomatic adolescents to diagnostic procedures and antimicrobial treatment to prevent a disease that usually manifests decades later. Its risk–benefit balance therefore requires more scrutiny than eradication undertaken for established *H. pylori*-related disease.

This balance becomes increasingly important as infection prevalence declines. Fewer infections are detected for a given number of individuals screened, while the relative importance of confirmatory testing, false-positive primary results, program costs, and antibiotic exposure increases. Population screening should therefore not be regarded as intrinsically beneficial simply because *H. pylori* is carcinogenic; its net value depends on local infection prevalence, gastric cancer risk, diagnostic performance, treatment effectiveness, and completion of the care cascade.

### 4.2. Antimicrobial Resistance and Stewardship

Antimicrobial resistance is a major consideration when eradication therapy is incorporated into population-based screening. In the Matsumoto school-screening program, clarithromycin resistance was detected in 41.1% of isolates from infected adolescents, yet susceptibility-guided treatment achieved a high initial eradication rate [17]. Takatsuki also demonstrated that treatment performance improved markedly after the first-line regimen was changed [15].

These findings argue against treating eradication therapy as a fixed component of screening. Local resistance patterns and observed treatment outcomes should be monitored, and regimens should be modified when effectiveness declines. This is particularly important in a public-health program because antibiotics are administered to asymptomatic individuals and the cumulative ecological consequences extend beyond individual eradication success.

### 4.3. Gut Microbiota After Eradication Therapy

The ecological consequences of eradication therapy are particularly relevant when antibiotics are administered to healthy adolescents. Adolescent-specific prospective data from Japan show measurable short-term changes in the intestinal microbiota. Gotoda et al. observed transient dysbiosis after eradication in teenagers, with species richness and evenness returning to pretreatment levels by two months, although compositional changes persisted in some individuals [20]. In a prospective multicenter study, Kakiuchi et al. likewise observed an immediate reduction in alpha diversity after vonoprazan-based therapy followed by recovery to the pretreatment condition at the time of eradication assessment [21].

Longer-term randomized adult data from Taiwan provide complementary evidence. Liou et al. showed that the magnitude and persistence of microbiota disturbance varied by eradication regimen and documented transient increases in antimicrobial resistance among intestinal bacteria, with substantial recovery over follow-up for several outcomes [22]. These adult findings should not be treated as adolescent-specific evidence.

Taken together, currently available data suggest that major disturbances in microbial diversity after eradication may be substantially reversible, but adolescent studies remain small and long-term clinical consequences are uncertain. Microbiota effects and antimicrobial resistance should therefore remain part of the risk–benefit assessment of population-based treatment.

### 4.4. Risk-Informed and Family-Based Approaches

As prevalence falls, risk-informed approaches may become increasingly relevant. Family information is one potential means of enriching the screened population because infection is commonly acquired within households and gastric cancer risk also clusters within families. In Yokosuka, 48.5% of infected students with available family information had at least one known *H. pylori*-positive relative [19].

However, this observation does not establish family-based screening as a substitute for population screening. Family infection status was incomplete, and many infected adolescents had no known infected relative. The updated ESPGHAN/NASPGHAN guideline supports noninvasive testing in children with a first-degree relative with gastric cancer in its European and North American context [11], but this recommendation addresses gastric cancer family history rather than household *H. pylori* positivity and should not be conflated with a Japanese family-based screening strategy.

Family-based and other risk-informed approaches are therefore best regarded as complementary options whose relative value should be tested prospectively as prevalence changes.

## 5. Lessons from Real-World Adolescent Screening Programs in Japan

### 5.1. Feasibility Across Different Regional Settings

More than a decade of school- and municipality-based *H. pylori* screening in Japan provides an important opportunity to evaluate adolescent screening under real-world conditions. Representative programs with sufficiently detailed published data include Saga Prefecture, Takatsuki City, municipalities in Akita Prefecture, Matsumoto City, Kyoto Prefecture, and Yokosuka City [14,15,16,17,18,19]. These programs are illustrative rather than exhaustive; the broader Japanese literature contains additional initiatives with heterogeneous designs [10].

Collectively, the programs demonstrate that organized adolescent screening is operationally feasible but that participation and confirmed infection prevalence vary substantially across settings (Table 1). Initial screening uptake ranged from approximately 60% in some municipality-based programs to more than 97% in Akita and Matsumoto [14,15,16,17,18,19]. These differences emphasize that implementation success in one region cannot simply be assumed in another.

### 5.2. Screening Should Be Evaluated as a Complete Care Cascade

Initial screening uptake alone is an insufficient measure of program success. Effectiveness depends on completion of sequential steps including participation, confirmatory testing, treatment initiation, eradication, and confirmation of treatment success. Attrition at any stage reduces the number of infected adolescents who complete the prevention pathway.

The Matsumoto program illustrates this distinction particularly clearly. Although 5178 of 5193 eligible students (99.7%) underwent initial urinary antibody screening, only 103 of 184 students with a positive primary result completed the recommended secondary evaluation [17]. Yokosuka likewise documented loss between primary screening and confirmatory UBT [19]. Long-term Kyoto data further showed that continued availability of screening did not guarantee sustained linkage to subsequent medical consultation [18].

These observations support evaluation of adolescent screening as a complete care cascade. At minimum, programs should separately monitor screening uptake, completion of confirmation, treatment initiation, first- and subsequent-line eradication success, adverse events, and confirmation of eradication.

### 5.3. Eradication Outcomes Are Modifiable Components of Program Performance

Real-world eradication outcomes have varied across Japanese programs and over time. In Saga, eradication was successful in 85.1% of treated students in the initial prefecture-wide report [14]. In Takatsuki, first-line success improved substantially after the program changed from a rabeprazole-amoxicillin-clarithromycin regimen to a vonoprazan-based regimen, and the final eradication rate after subsequent therapy was high [15]. In Matsumoto, susceptibility-guided initial therapy achieved 96.5% eradication among evaluated students despite a clarithromycin resistance rate of 41.1% [17].

Kyoto also documented improvement in eradication success after treatment strategies changed during long-term implementation [18], whereas Yokosuka showed relatively low first-line success but substantially higher overall success after second-line treatment [19]. These results should not be interpreted as direct comparisons of therapeutic efficacy because study periods, regimens, resistance patterns, and follow-up differed. Rather, they show that eradication performance is a modifiable component of a screening program and should be periodically reassessed.

### 5.4. Declining Prevalence Changes the Screening Environment

Successive adolescent cohorts screened in Japan have shown a substantial decline in *H. pylori* prevalence. The Kyoto program provides a particularly clear recent example, with confirmed infection decreasing from 4.7% in 2015 to 1.5% in 2023 [18]. This observation is consistent with the broader global and birth-cohort decline in *H. pylori* prevalence [2].

Importantly, these temporal trends should not be interpreted as evidence that screening programs themselves caused the decline in *H. pylori* prevalence. Screening documents the changing epidemiological environment; the decline is more plausibly understood within broader changes in childhood acquisition, socioeconomic conditions, and household environments [2].

Declining prevalence nevertheless has direct consequences for screening policy. Screening yield falls, the predictive performance of primary tests changes, and the relative importance of confirmatory testing, program costs, and antibiotic exposure increases. Thus, a strategy that was reasonable under one epidemiological condition should not be assumed to remain optimal indefinitely.

Taken together, Japanese programs demonstrate both the feasibility and the limitations of population-based adolescent screening. Participation, diagnostic completion, antimicrobial resistance, treatment effectiveness, and infection prevalence vary across place and time. The central policy question is therefore not simply whether adolescent screening can be implemented, but how its target population, diagnostic pathway, treatment strategy, and evaluation framework should evolve.

## 6. Toward Adaptive Population-Based Prevention

Population-based adolescent *H. pylori* screening should not be regarded as a fixed intervention that remains appropriate indefinitely once introduced. The Japanese experience shows that infection prevalence, diagnostic performance, antimicrobial resistance, treatment effectiveness, participation, and retention across the care cascade can all change. We therefore propose an adaptive approach in which screening strategies are periodically reassessed according to contemporary epidemiological and healthcare conditions.

### 6.1. What Should Be Adapted?

Adaptation may occur at several levels. The target population may shift toward more risk-informed approaches if prevalence becomes sufficiently low; diagnostic algorithms should be reassessed as the predictive value of primary screening changes; eradication regimens should respond to local resistance and observed treatment effectiveness; and program delivery should be modified when attrition is concentrated at a particular stage of the care cascade.

Adaptation does not necessarily mean reducing screening intensity. Persistent regional gastric cancer risk, local *H. pylori* prevalence, or inequalities in access to healthcare may continue to favor population-based approaches in some settings. Decisions should therefore be based on local evidence whenever reliable data are available.

### 6.2. International Context: Different Strategies for Different Epidemiological Settings

International experience further illustrates why a single *H. pylori* prevention strategy is unlikely to be appropriate for all populations. Japan has developed an adolescent school-based model that combines early intervention with organized access to defined birth cohorts [9,10].

A contrasting population-based model has been implemented on the Matsu Islands of Taiwan, a community with historically high *H. pylori* prevalence and gastric cancer risk. Repeated community-based screening and eradication in adults achieved high population coverage, reduced *H. pylori* prevalence from 64.2% to 15.0%, and was associated with a 53% lower gastric cancer incidence than expected from the historical control period [23]. These findings provide important long-term evidence for population-based eradication, but the target age, baseline prevalence, and underlying cancer risk differ substantially from Japanese adolescent programs.

In contrast, the updated ESPGHAN/NASPGHAN guideline reflects a more selective pediatric approach in Europe and North America, including noninvasive testing for children with a first-degree relative with gastric cancer rather than general population screening [11]. These different policies should not be viewed as contradictory: they arise from different epidemiological and healthcare contexts. The relevant question is therefore not which strategy is universally correct, but which strategy provides the greatest net benefit in a given setting.

### 6.3. Cost-Effectiveness in a Declining-Prevalence Population

Declining infection prevalence raises an unresolved economic question: at what point does universal adolescent screening cease to provide sufficient additional benefit relative to its costs and potential harms? As prevalence decreases, more individuals must be screened to identify each infection, and the relative contributions of confirmatory testing, administration, and antibiotic exposure become more important.

Health-economic analyses have supported *H. pylori* screening and eradication in selected adult Japanese populations [24], but these findings cannot be directly extrapolated to school-based adolescent programs. The revised Japanese guideline also highlights the limited direct comparative evidence for public-health testing of asymptomatic minors [9]. Contemporary economic evaluations should therefore incorporate adolescent prevalence, diagnostic performance, attrition across the care cascade, eradication effectiveness, antimicrobial resistance, and the decades-long time horizon to gastric cancer prevention.

No evidence-based prevalence threshold can currently be proposed at which Japan should automatically move from universal adolescent screening to targeted testing. Defining such thresholds is an important priority for future research.

### 6.4. From Universal to Risk-Informed Screening?

If universal screening becomes less efficient, potential alternatives include geographically targeted screening, family-history- or household-informed approaches, and combinations of epidemiological risk factors. Current evidence does not establish that these approaches can identify all infected adolescents or reproduce the broad access provided by school-based screening [9,19]. A transition to targeted screening should therefore be evidence-driven rather than assumed to be an inevitable consequence of declining prevalence.

### 6.5. What Should Future Programs Measure?

Future evaluations should move beyond reporting only the number screened or the prevalence of positive primary tests. A minimum standardized outcome set should include: (1) eligible population and screening participation; (2) primary screening positivity; (3) completion and result of confirmatory testing; (4) treatment initiation; (5) first- and subsequent-line eradication success; (6) adverse events; (7) antimicrobial resistance where available; and (8) confirmation of eradication. Program costs and reasons for attrition should also be recorded whenever feasible.

Long-term follow-up is ultimately required to determine whether adolescent screening reduces gastric cancer incidence and whether its benefits justify the cumulative costs and potential harms of population intervention. Because those outcomes will take decades to mature, intermediate measures of program performance should be standardized prospectively.

**Figure 1:**
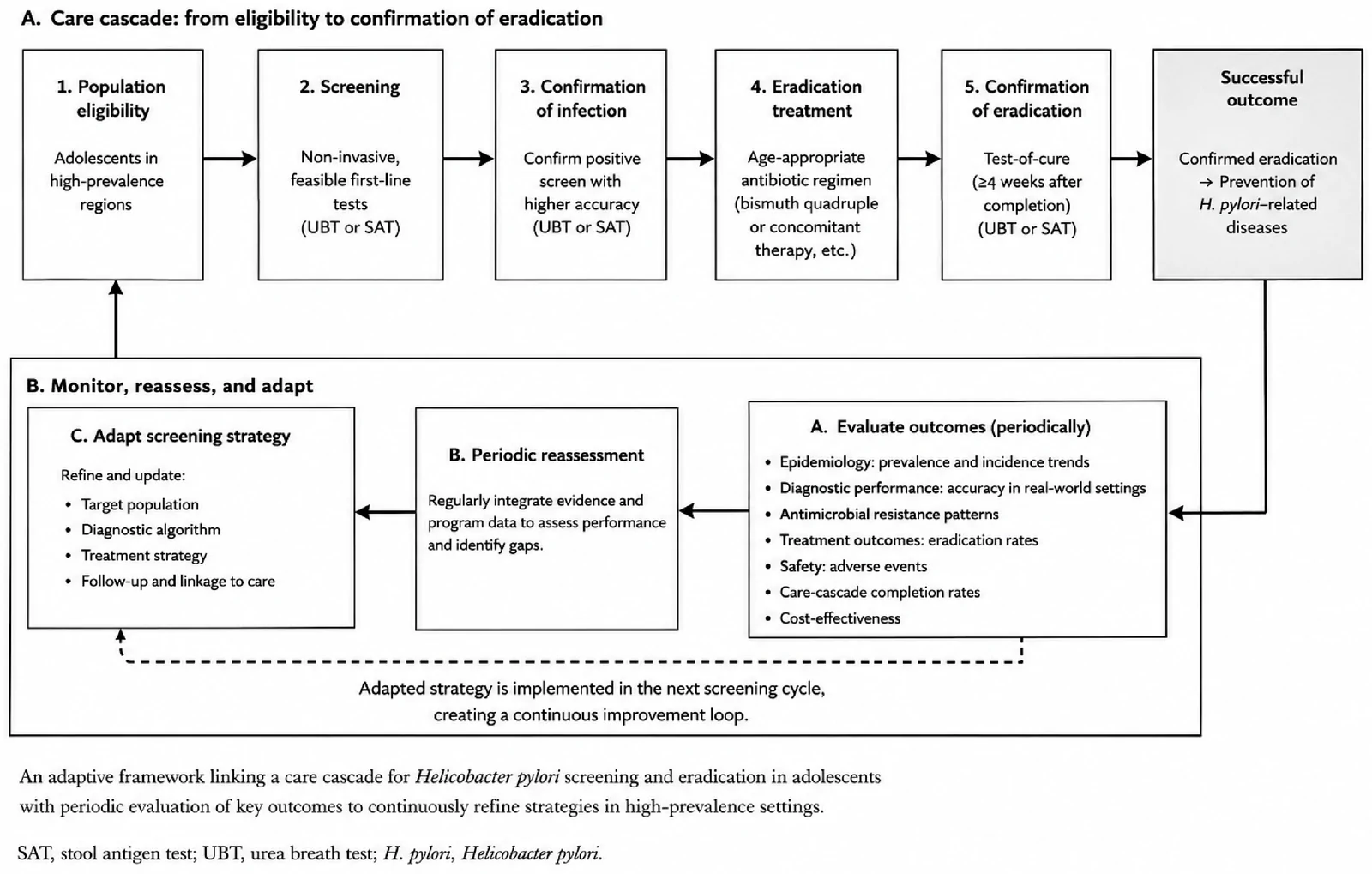
Proposed adaptive framework for population-based adolescent *Helicobacter pylori* screening. Screening is conceptualized as a care cascade extending from population eligibility through confirmation of successful eradication. Program performance should be periodically reassessed using epidemiological, diagnostic, therapeutic, safety, implementation, and economic indicators, allowing the target population, diagnostic algorithm, and treatment strategy to be modified as conditions change. Numbers indicate sequential steps in the care cascade; arrows indicate progression through the cascade and feedback for subsequent adaptation.

The proposed adaptive framework is summarized in Figure 1. Screening is conceptualized as a care cascade extending from eligibility through confirmation of successful eradication, with periodic reassessment of epidemiology, diagnostic performance, antimicrobial resistance, treatment outcomes, safety, care-cascade completion, and cost-effectiveness.

## 7. Conclusions

Adolescence represents a strategically favorable window for population-based *H. pylori* screening in Japan, combining an opportunity for eradication before advanced precancerous gastric changes develop with the practical advantages of school-based access to defined birth cohorts. More than a decade of Japanese experience demonstrates that organized adolescent screening is feasible while also revealing substantial variation in participation, diagnostic completion, antimicrobial resistance, treatment outcomes, and retention across the care cascade.

However, direct evidence that adolescent screening reduces future gastric cancer incidence or mortality is not yet available, and the balance of benefits, harms, and costs will change as *H. pylori* prevalence continues to decline. Screening should therefore be viewed as an adaptive prevention strategy rather than a fixed policy. Continued evaluation of local epidemiology, diagnostic performance, treatment effectiveness, safety, antimicrobial resistance, care-cascade completion, and cost-effectiveness will be essential to determine when population-based screening remains appropriate and when more targeted approaches should be considered.

## Figures and Tables

**Table 1:** Representative Population-Based Adolescent *Helicobacter pylori* Screening Programs in Japan: Screening Pathways and Implementation Outcomes.

Region/Primary Study	Study Period/Target Population	Initial Screening Participation	Screening → Confirmation	Infection Findings	Eradication Outcomes	Key Implementation Findings
Saga Prefecture [14]	2016–2018; third-year junior high school students	21,042 screened; 83.1%	Urinary antibody → stool antigen test	660/21,042 (3.1%) confirmed infection	501 treated; 85.1% success	AE 20/501 (4.0%); no serious complications; participation increased over time
Takatsuki City, Osaka [15]	2014–2017; second-year junior high school students	8067/13,055 (61.7%)	Urinary antibody → 13C-UBT	461 primary positive; 426 UBT; 206 confirmed (2.6% of screened)	First-line success improved from ~46% with RAC to 83.8% with VAC; final eradication 98.5%	Regimen change markedly improved treatment performance; no severe AE reported
Yurihonjo/Nikaho, Akita [16]	2015; second-/third-year junior high school students, 13–15 y	1765/1813 (97.3%)	Urinary antibody → 13C-UBT	96 primary positive; 90 UBT; 85 confirmed (4.8% of screened)	NR in primary screening publication	93.7% of primary-positive students completed confirmatory testing
Matsumoto City, Nagano [17]	2007–2017; high-school students	5178/5193 (99.7%)	Urinary antibody → EGD, histology, culture	184 primary positive; 103 secondary evaluation; 90 confirmed	Susceptibility-guided initial eradication 82/85 (96.5%) among evaluated students	CAM resistance 41.1%; AE 11/85 (12.9%); one rash required hospitalization; secondary-stage attrition
Kyoto Prefecture [18]	2015–2023; first-year high-school students	50,031 targeted; annual consent 59.9–83.9%	Urinary antibody → stool antigen test	Confirmed infection 4.7% (2015) → 1.5% (2023)	PAC 76.5%; PAM ≥93.8%; >96% after later probiotic co-administration strategy	Medical consultation among infected students declined markedly over time; no serious AE reported
Yokosuka City, Kanagawa [19]	2019–2021; second-year junior high school students	6270/9879 (63.5%)	Urinary antibody → UBT	266 primary positive; 231 UBT; 73 confirmed (1.2% of screened)	First-line 37/70 (52.9%) PP; overall after second-line 94.7% PP	35/266 primary-positive students did not complete UBT; mainly mild AE; 48.5% with family data had ≥1 known infected relative

Abbreviations: AE, adverse event; CAM, clarithromycin; EGD, esophagogastroduodenoscopy; NR, not reported in the cited primary publication; PAC, proton pump inhibitor-amoxicillin-clarithromycin; PAM, proton pump inhibitor-amoxicillin-metronidazole; PP, per-protocol; RAC, rabeprazole-amoxicillin-clarithromycin; UBT, urea breath test; VAC, vonoprazan-amoxicillin-clarithromycin. Note: Primary screening positivity and confirmed infection are reported separately whenever available. Eradication outcomes should not be directly compared across programs because study periods, treatment regimens, resistance patterns, outcome definitions, and follow-up procedures differed.

## Data Availability

No new data were created or analyzed in this study. Data sharing is not applicable to this article.
